# Reviews

**Published:** 1922-12

**Authors:** 


### REVIEWS OF BOOKS.

**LOOKING ON.** By Jimmy Howcroft. (Second Edition; 2s. 6d.)

Mr. Howcroft, a permanently disabled airman, who has been unable to move even his arms and legs since 1916, began his career as a poet in the wards of the London Hospital. Despite his constant pain, he found an amanuensis in his nurse, who wrote from his dictation the verses that have now run into a second edition. Thanks to the welcome that his book received, he now lives at Little Forest Cottage, Liphook, where he publishes his work, and from which copies may be obtained. If the demand for this second edition, which has been revised, enlarged, and illustrated by a portrait, is repeated, the poet will be able to add the cares of poultry farming to his already busy existence. We suppose that it is natural to find courage and cheerfulness most conspicuously in those who have to build their lives upon them. Mr. Howcroft is certainly the embodiment of both. In one poem he takes an aeroplane to fairyland, and the line

"We'll cut a window in the sky,"

has a professional, no less than a poetic, quality. It suggests mechanical flight. Indeed, memories of flying, doctors, and nurses run through many of the verses in the book. Up in the air, or under the sea, anywhere out of the world, in fact, is a pleasure to his imagination, though the robust delights of harvesting, beer, and summer sun are also celebrated. Once or twice poetry is very near to him, and if he could sustain his glider a little longer it would be possible to quote a completely achieved poem. Such a beginning as

Oh, my home is in the valley,
But my spirit haunts the hill, or the following lyric, called "Silence," are achieved things. "After the Toil" and "A Dream" are two of the best, and curiously akin to Mr. W. H. Davies' manner, whose exquisite simplicity is unapproached by any other contemporary poet. Of the three ingredients of poetry—substance, diction, and metre —Mr. Howcroft often has the first, and sometimes the second or third. He has an eye, feeling, and, usually, artistic sincerity. His volume is worth reading for its own sake, and every reader who procures it will not only have that pleasure, but the curious delight of helping to turn a poet into a poultry farmer. We hope, however, that care for diction and metre will not be wholly diverted to his fowls.—O.B.

---

**A STUDY OF INTESTINAL STASIS.** By J. C. Wyatt. (John Bell, Sons & Danielsson, Ltd.; 1s. net.)

In his introduction to this pamphlet the author disarms anticipated criticism of its form and manner of publication by the naïve confession that he has been unsuccessful in his attempt to get it accepted by a medical journal. From his determination, his emphasis and his frequent use of italics it is obvious that his convictions are strong. He gives a new presentation of the familiar doctrine that intestinal stagnation is the cause of every ill. The author goes farther than most in considering that these seeds of ill health begin to grow in early childhood. For chronic constipation, overt or concealed, he blames the pelvi-rectal junction, and has devised an operation which should overcome the difficulty. Unfortunately, no results of practical experience are advanced to support or dispute his claim, and unless some open-minded surgeon will put us in possession of these, Dr. Wyatt's ideas must remain only theoretical.—C. E. S.

---

**THE INTENSIVE TREATMENT OF SYPHILIS AND LOCOMOTOR ATAXIA BY AACHEN METHODS.** By Reginald Hayes. 4th Edition, revised. (Baillière, Tindall & Cox; 4s. 6d. net.)

This little book is a reasoned plea for the adoption of inunction treatment in all stages of syphilis. The value of salvarsan and its substitutes is recognised, but the limitations of these drugs are pointed out, and their apparent inability to prevent late manifestations of the disease is emphasised. The author is an enthusiastic believer in the value of the Aachen method of repeated courses of inunction of mercury, combined with the use of sulphur internally and externally. He discusses impartially the difficulties and drawbacks of this treatment and disposes of objections often raised. Success depends upon attention to details; these are clearly described. Unfortunately this method does not lend itself to general use in venereal clinics; it is too exacting in time and technique, and is not so impressive as intravenous injection. Its use in private practice, however, can be strongly advocated. This book is an excellent guide.—C. E. S.

**DOSAGE TABLES FOR DEEP-THERAPY.** By Prof. F. Voltz. Revised and edited by R. Morton, M.D. (Wm. Heinemann, Medical Books, Ltd.; 10s. 6d. net.)

It is important that those who employ dangerous remedies should know their "doses." Such knowledge it is the business of our examiners to enforce upon the would-be medical man. The use of X-rays is as dangerous as that of many powerful drugs, yet dosage is largely left to the choice of the individual administrator. This little book is an attempt to put the practice of radio-therapy upon a firmer footing. Its appeal can only be to a limited circle, many of whose members have already worked out their own technique, but for the less experienced it presents in a convenient form means of estimating dosage which if adhered to should ensure adequate yet safe radiation.—C. E. S.

---

**SAFE MARRIAGE.** By Ettie A. Rout. (Heinemann; 3s. 6d. net.)

Miss Rout has the courage to undertake a task which should long ago have been performed by a member of the medical profession. She speaks with a plainness that proclaims her sincerity, while it is a rebuff to the prurient. Her little book should go far to answer the questions which so many patients ask and so many more would like to ask about the essential physical factor of marriage.—C. E. S.

**SUGGESTION AND MENTAL ANALYSIS.** By William Brown, M.A., M.D., D.Sc., M.R.C.P. Pp. 165. (University of London Press; 3s. 6d. net.)

Any work by Dr. William Brown commands respectful attention. But we must confess to a feeling of disappointment on reading his latest book, and of doubt as to whether it will enhance his high reputation by its publication. The book is intended to give an elementary and non-technical account of the relation between suggestion and auto-suggestion on the one hand, and mental analysis on the other. Dr. Brown believes that they can be harmonised, but we doubt this, and do not think that he has proved his thesis. Both methods have their uses, and none but a fanatic would refuse to make use of suggestion in suitable cases. But Freudian analysis is altogether different from suggestion. The book contains an accurate and sympathetic account of the Freudian theory, but it is far too short to be of any value to the reader who desires to have the theory of psycho-analysis explained. There is also an interesting but equally short description of the process of suggestion, and of M. Coué's practice. Dr. Brown, however, points out that specialists in neurology and psychotherapy have employed similar methods of treatment for many years. Technical readers will be surprised to learn that Dr. Brown appears to treat melancholia by suggestion. The last quarter of the book is of philosophical nature, and contains an account and a criticism of Bergson's system. But the reader who has some acquaintance with philosophy will find Dr. Brown's treatment of this subject tantalisingly brief and inadequate. And we venture to think that a reader who is ignorant of philosophy will find it incomprehensible. Determinists will not be disposed to accept Dr. Brown's view of "will," and they would demur to the statement that they, as well as libertarians, "over-emphasise the distinction of the will and the deed." We are told that parts of the book were delivered in the form of extempore lectures. It would seem that Dr. Brown presupposed a certain standpoint on the side of his audience, for he uses the words "soul" and "faith," with no explanation of the sense in which he employs these most controversial expressions. And he speaks of "good" and "bad" suggestions, without any reference as to the standard which is to be used in making these comparisons. There is no index, a defect which we hope Dr. Brown will correct should a subsequent edition be issued.

---

**TRANSACTIONS OF THE SECOND INTERNATIONAL CONFERENCE OF THE INTERNATIONAL UNION AGAINST TUBERCULOSIS, London, July, 1921.** Pp. 264. (Adlard & Son & West Newman, Ltd.)

Among the principal aspects of the vast problem of tuberculosis that were touched upon at the Second International Conference of the International Union against Tuberculosis held in London in 1921, the mode of the diffusion of the disease throughout the various races of the world was the one, perhaps, around which the greatest interest centred. If we are certain as to the bacterial origin of tuberculosis, we are not so sure in regard to other and lesser known factors which determine its spread. As Prof. Calmette pointed out, the recently acquired knowledge of the elimination of the tubercle bacillus in the glandular or intestinal excretions of apparently healthy people further complicates our efforts towards prophylaxis. Many speakers emphasised, and rightly so, the need for protection against the bacillus, especially in the case of young children who are most susceptible to the disease. The training of those who intend to become specialists in tuberculosis and the need for caution in dealing with any new medicament announced for the treatment of consumption were among other engrossing topics that claimed the attention of the members of the Conference. Every paper contained in this volume will be read with interest and profit by all who have the cause of the prevention of tuberculosis at heart.—G. N. M.

---

**THE ROYAL DEVON AND EXETER HOSPITAL.** By J. Delpratt Harris, M.D. (Exeter: Eland Bros.; 5s. net.)

This chronicle, edited by one of the consulting surgeons to the hospital, is something between a formal history and a record of the minute books from 1741 to the present time. In this respect it is somewhat tantalising, for we wish to know more of the men and the work than the editor has been able to give us, and are at the same time grateful for the pen-portraits of different members of the staff which enliven the diary-form in which these notes are presented. A useful introductory chapter gives a survey of the development of voluntary hospitals which marked the eighteenth century, and the following details of the growth of the hospital at Exeter are typical of the work accomplished and the social conditions elsewhere. The hospital has always been rich in portraits of its chief medical men, often by artists of repute; and those illustrated, with the pen-portraits collected or supplied by Dr. Delpratt Harris, give interest and liveliness to his narrative. Among many details which show the condition of the hospital at different periods since its foundation in 1741, we note that the board room floor was sanded till 1745, that walls were papered in 1750, that the hospital for a long time baked its own bread and brewed its own beer, that as early as 1756 separate wards were started for operation cases, and that Mr. Ralph Allen, an early chairman, was believed to be the original Squire Allworthy in "Tom Jones." He invented a cross-country postal service, amassed a great fortune, and was the friend of Alexander Pope. In 1767 a skeleton was ordered for the use of the hospital, and in 1779 an apparatus for resuscitating the apparently drowned; they probably needed it, from the risks of repairing wells and those attending the shipping then abundant on the river. The first secretary was Mr. William Chapple, appointed in 1743, who died in 1780: an exquisite master of caligraphy, whose books and minutes are still a joy to behold, and must be as beautiful a monument to their writer as are the portraits in the board room, in which the work of Opie is represented. The pumping of the water was long a duty expected of the patients. The finances on the whole were so satisfactory that the committee in 1793 apologised for investing a portion of its funds, and disclaimed all idea of creating a permanent endowment. In 1796, however, 43 beds were closed, but this crisis was temporary. Elections to vacancies on the honorary staff brought in temporary subscribers anxious to support at the election the candidate of their choice. Mr. Coggan, who succeeded Mr. Chapple, was also an adept with the pen, but, not being so accurate a book-keeper, eventually resigned. Of the long list of interesting surgeons there is no space to speak, but Dr. Harris puts in a good word for the old type of nurse whose homely virtues are nowadays obscured by the vices of Mrs. Gamp and other notorious delinquents. Mrs. Lott, the famous matron, was appointed in 1824, and it is tempting to linger on the personality of herself, of Miss Children, and others who recall the past so vividly. The old type of nurse lingered on till 1889. The whole is an honourable and interesting story, still happily far from complete. —O. B.

---

**FUNCTIONAL NERVOUS DISORDERS: THEIR CLASSIFICATION AND TREATMENT.** By Donald E. Core, M.D.Manch., M.R.C.P. Pp. xiii, 371. (John Wright & Sons; 25s. net.)

The time-honoured distinction between functional and organic disease, as applied to the nervous system, still holds good, in spite of the considerable amount of research in neurology that has taken place in recent years. As the author of this book rightly states, however, a diagnosis of sympathetic functional nervous disorder is not concise enough "for clinical purposes, and a further division on psychical grounds is necessary." He accordingly divides this large class of affections into two groups: (1) regressive, comprising the hysterias, and (2) progressive, including (*a*) the dysthymias, centrifugal and centripetal, and (*b*) the mnemoneuroses. The question of hysteria is fully discussed in some 90 pages. Its essential associates are those of a faulty environment of determination, a lack of emotional control, and a certain amount of psychical dissociation. The cardinal principles of treatment in all hysterical manifestations are to render the disability a source of vexation to the patient, the adoption of a non-punitive attitude, "the prevention of the thoroughly dangerous atmosphere of self-pity," the creation of a detached atmosphere, distasteful to the patient, and the replacing of the feeling of defiance—a characteristic attitude of the hysterical patient—by the desire to please. The differential diagnosis of the various functional nervous disorders is clearly set forth, and a scheme of case-taking is given which, if generally adopted, would materially simplify the classification of the patient's disorder, and thus render the treatment more readily applicable. The reader will be disappointed if he expects to find an account of modern psycho-analytical technique, which, as the author states, amounts "to little more than detailed case-taking," nor will he find "Couéism" mentioned in this volume, which will be welcomed alike by psychologists and students of neurology.—G. N. M.

**THE STUDENT'S POCKET PRESCRIBER.** By David Mitchell Macdonald, M.D., F.R.C.P.E. 7th Edition. (E. & S. Livingstone, Edinburgh; 35s. net.)

Materia Medica is a bugbear to most students, and how and what to prescribe are questions that the newly qualified are apt to find very difficult. Most of us must at times have wished that we had a pocket *vade mecum* upon which to rely. That this little volume has reached a seventh edition is proof that it supplies a want. It affords in a form small enough for the waistcoat pocket a list of drugs and doses, with useful hints upon prescribing and safe prescription for most common symptoms. Both imperial and metric systems are used. With this book within reach even the least experienced practitioner should be able to resist the ever-present temptation to rely upon synthetic drugs and stock mixtures. The author has done well what he set out to do. He gives no scope for hostile criticism, but the description of pharmacopeial changes on page 5 is ambiguous; it is not quite clear whether it is the old or the new strength of drugs which is given in the last column. A very useful little book.—C. E. S.

---

**THE ELECTRICAL ACTION OF THE HUMAN HEART.** By Augustus D. Waller. (University of London Press; 7s. 6d. net.)

Dr. Waller's pioneer work upon this subject is so well known that anything that he writes is sure of finding an appreciative public. The present volume is based upon a course of lectures delivered in 1913. While the clinical aspect of the subject is not ignored, the physiological aspect is discussed more fully. The author finds himself unable to accept all the interpretations which are commonly offered of variations in electrocardiographic records; he holds that some of these to which a pathological significance is often attributed can be explained on the grounds of physiological variation consistent with health. The book is the product of an original mind, for which truth is more important than "authority." While the value of this study is merely indirect to the general physician, it is one which physiologists and cardiologists cannot afford to ignore. Much of it is highly technical, but there can be no question of the need for such a review of this aspect of physiology.—C. E. S.

---

**KETTLE SONGS.** By Habberton Lulham. (Hove, Combridges; 3s. 6d. net.)

The fact that a large number of verses included in this volume appeared originally in "Punch" gives a clue to all not familiar with the author's other volumes of the aim and quality of his work. Mr. Lulham has an undoubted metrical gift which plays happily on the border of poetry and humour. It is light verse of accomplished quality, though not free from those occasional lapses which, for the moment, make a lover of poetry feel that light verse is an abomination. Such poems as "On the Safe Side," "A Footprint in Spring," "Jewels," show him at his best, while "A Singular Case" displays that interior humour, the humour of idea, which is perhaps the only real place where humour is admissible in poetry. All who care for light verse can be recommended to read this book.—O. B.

